# GeriHITSS$: A Brief, Reliable Tool for Identifying Elder Abuse in Clinical Practice

**DOI:** 10.1177/21501319261478038

**Published:** 2026-08-08

**Authors:** Sanjna Bhatia, Sarah B. Woods, Leo Gonzalez, Chance R. Strenth, Alejandro Martinez, Victoria Udezi, Amer Shakil

**Affiliations:** 1 The University of Texas Southwestern Medical School, Dallas, TX, USA; 2 12334 Department of Family and Community Medicine, The University of Texas Southwestern Medical Center, Dallas, TX, USA

**Keywords:** elder abuse, community health/public health, screening, geriatrics, primary care

## Abstract

**Objective:**

Elder abuse is a prevalent but underrecognized public health issue. This pilot reliability study aimed to develop the GeriHITSS$, a six-item screening questionnaire to identify forms of abuse experienced by geriatric populations, adapted from the well-established, valid, and reliable HITS screening tool.

**Methods:**

A cross-sectional study was conducted including 199 primary care patients aged 65 and over recruited from two family medicine clinics in the Southwestern United States between April and December 2023. Participants completed the GeriHITSS$, which assessed physical, emotional, verbal, sexual, and financial abuse.

**Results:**

The study sample had an average age of 72.5 years (SD = 5.77) and were predominantly female (60.1%). Emotional abuse was the most frequently reported, while physical abuse, sexual abuse, and financial abuse were less frequent. The GeriHITSS$ demonstrated acceptable internal reliability (α = .721). Scale scores were not significantly linked to participant sex, race, nor age.

**Conclusions:**

GeriHITSS$ is a reliable screening tool to detect elder abuse in outpatient primary care settings. Incorporating routine screening may enhance early detection and support interventions to address this critical health concern.

## Introduction

Elder abuse is a significant yet underrecognized public health concern affecting millions of older adults worldwide.^1^ Elder abuse is defined as a negligent or intentional act that causes harm or a serious risk of harm to an older adult.^2^ Studies prior to the COVID-19 pandemic estimated that one in six individuals globally over the age of 60 have experienced some form of elder abuse.^1^ This proportion worsened for older Americans during the COVID-19 pandemic, when approximately one in five older adults reported abuse.^3^ Despite its detrimental consequences, including physical harm, psychological distress, and increased mortality, elder abuse often goes undetected in clinical settings due to limited validated screening tools.^4^ Health care professionals frequently miss opportunities for early intervention, as routine screening for elder abuse remains inconsistent across outpatient clinics, nursing homes, and emergency departments.^5^

**Figure 1.**
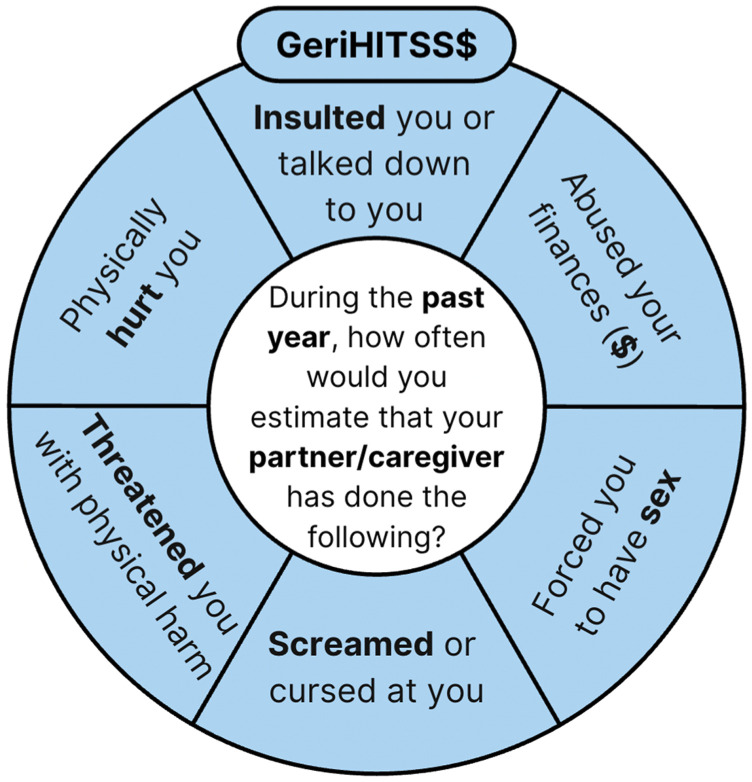
GeriHITSS$ tool diagram

Current screening instruments for elder abuse are limited in their ability to comprehensively assess risk in the outpatient setting, particularly regarding financial and sexual abuse—two prevalent yet often overlooked forms of abuse among older adults. Prior reviews of elder abuse screening tools have highlighted substantial variability in instrument content, psychometric performance, and clinical applicability, with many tools lacking comprehensive coverage of abuse domains or validation across diverse care settings.^6^ While newer instruments such as the Hospitalized Elder Abuse Questionnaire (HEAQ) have demonstrated promising psychometric properties in inpatient populations, there remains a need for brief, reliable screening tools specifically designed for outpatient use.^7^ This gap underscores the importance of developing and evaluating instruments that can efficiently identify multiple forms of elder abuse in routine ambulatory clinical practice. To address this gap, this study aimed to develop the self-report Geriatric Hurt-Insult-Threaten-Scream-Sex-Steal (GeriHITSS$) tool*,* a six-item questionnaire designed specifically for use in geriatric populations (Figure 1). GeriHITSS$ is an adapted version of HITS screening tool.^8^ The original HITS screening tool was developed by one of the research team members of the current study. HITS is a consistent, valid, and reliable measure used to assess family and intimate partner violence and abuse.^8^ The HITS questionnaire has previously been validated in pediatric, adolescent, and adult populations.^8-12^ By comparing the performance of the 6-item GeriHITSS$ to the previously validated 4-item HITS screening tool for adults, this pilot reliability study aims to establish the reliability of GeriHITSS$ and evaluate its usefulness as a practical screening tool for identifying older adults at risk of abuse in family medicine outpatient settings, thereby supporting earlier identification and intervention.

## Methods

This study was approved by the medical center’s Institutional Review Board. Our study aim required the collection of sensitive information on abuse experiences in a vulnerable aging population. Thus, participants solely provided verbal consent (and written documentation of informed consent was waived by the IRB) to allow participants to answer anonymously and limit the collection of information that could be used to identify the participants. Approval to waive documentation of signed informed consent also allowed us to avoid registering patients as research participants in a study on geriatric abuse in their electronic medical record, which may have been accessed by family members or caregivers who were engaged in the abuse being reported. Participants reviewed a study information sheet with the study team. Once they confirmed their understanding of the study and any remaining questions were addressed, each eligible participant was asked to provide verbal informed consent that was documented by the study team.

### Study Design and Participants

A cross-sectional design was utilized to assess the reliability of the GeriHITSS$ screening tool in identifying abuse among geriatric primary care patients. Participants were recruited from two family medicine outpatient clinics associated with an academic medical center in the Southwestern United States between April and December 2023. Eligibility criteria included individuals aged 65 years and older, who spoke either English or Spanish, with no previously documented abuse in their electronic medical record and achieved a Mini-Cog score of 5. Permission to use the Mini-Cog for this study’s research purposes was granted by the developer, allowing the instrument to be used to evaluate participants’ eligibility. Participants with Mini-Cog scores less than 5 were excluded from the study.^13^ A total of 216 participants were screened with the MiniCog and 199 participants were included in the study, with missing data reported for select variables.

### Data Collection

Survey administration occurred during routine clinical visits, with responses recorded through REDCap.^14,15^ Research personnel approached patients either before or after their scheduled clinic visit, depending on provider preference, and asked if they wanted to participate in the study. Family members were asked to leave the room during the survey. If they preferred to stay, they were not permitted to assist or interact with the participants during the survey. In line with previous methods of developing geriatric screening tools, a MiniCog screener was administered to the participants to assess cognitive function.^13,16^ Participants then completed the six-item GeriHITSS$ questionnaire, a self-report measure in which participants independently reported the frequency of experiences related to emotional, physical, sexual, and financial abuse (Table 1).

**Table 1.** GeriHITSS$ Tool

During the last year, how often would you estimate that your partner/has done each of the following?	Rarely 1pt	Sometimes 2pt	Fairly often 3pt	Frequently 4pt	Never 0pt
Physically HURT you	​	​	​	​	​
INSULTED you or talked down to you	​	​	​	​	​
THREATENED you with physical harm	​	​	​	​	​
SCREAMED or Cursed at you	​	​	​	​	​
Forced you to have SEX	​	​	​	​	​
Abused your FINANCES($)	​	​	​	​	​

### Measures

The original HITS screening tool includes four items, assessing physical abuse (Hurt) and verbal abuse (Insult, Threaten, Scream).^8,9^ Item responses utilize a 5-point Likert scale ranging from 0 (*never*), indicating no experience of abuse, to 4 (*frequently*). Scale scores are calculated via summation. The HITS screening tool is well-established, internally reliable, and valid for use with adult and pediatric patient populations. The GeriHITSS$ and HITS were developed by one of the authors of the current research team and were used with the authors’ permission for the purposes of this study.

This study adapted the HITS to include two additional items assessing sexual abuse and financial exploitation, resulting in the GeriHITSS$. These two additional items utilized the same Likert score as the original four HITS items. Scale scores were similarly calculated via summation of item responses, ranging from 0 to 24. The measure was administered in both English and Spanish, and the Spanish version HITSS$ was adapted to Spanish for this study.

### Demographic Covariates

Participant characteristics were examined including age, sex (0 = *male,* 1 = *female*), and race (0 = *White,* 1 = *Other*). Race was coded as a dichotomous variable to explore scale scores for White and non-White individuals, primarily due to a significant portion of the sample identifying as White (47.2%).

### Statistical Analysis

Descriptive statistics were used to summarize participant characteristics and response distributions. Internal consistency of the GeriHITSS$ and HITS tool was assessed using Cronbach’s alpha. Mean comparisons were conducted to examine differences in scale scores linked to sex and race. Pearson correlations were used to examine the relationship between age and GeriHITSS$ scores. Independent samples t-tests and effect size calculations were conducted to assess differences in GeriHITSS$ scores across gender and racial groups. Individuals with missing cases were listwise deleted from all analyses.

## Results

Demographic characteristics of the sample are presented in Table 2. The sample was mostly female (60.1%), English-speaking (95.0%), and had an average of 72.5 years of age (SD = 5.77). Our sample was racially diverse, including individuals who identified as Asian (3.5%), Black (29.1%), Hispanic (15.6%), White (47.2%), and Biracial (1.0%). Missing data were minimal among the 199 included participants (Table 3).

**Table 2.** Participant Demographics (HITSS$ Survey, N = 199)

Variable	N (%)
Gender	
Male	80 (39.4%)
Female	122 (60.1%)
Race	
Asian	7 (3.5%)
Black/African American	58 (29.1%)
Hispanic	31 (15.6%)
White/Caucasian	94 (47.2%)
Biracial	2 (1.0%)
Other	5 (2.5%)
Age	
Range	65-93
Average	72.5
Median	72
Marital Status	
Single	36 (18.1%)
Divorced/Separated	39 (19.6%)
Married	91 (45.7%)
Widowed	32 (16.1%)
Has Children	
Yes	164 (82.4%)
No	28 (14.1%)
Average Number of Children	2.62
Health Insurance	
Medicaid	10 (5.0%)
Medicare	163 (81.9%)
Private Insurance	23 (11.6%)
No Insurance	1 (0.5%)
Language	
Spanish	10 (5.0%)
English	189 (95.0%)

*Note.* Not all percentages total 100% because some responses were missing.

**Table 3.** Descriptive Statistics and Abuse Reporting Among Older Adults (N = 199)

Abuse type/Measure	Valid responses (n)	None reported	Some/Any level reported
Physical Abuse	190	184 (96.8)	6 (3.2)
Emotional Abuse – Insults	189	155 (82.0)	34 (18.0)
Emotional Abuse – Threats	188	184 (97.9)	4 (2.1)
Verbal Mistreatment (Screaming/Cursing)	191	174 (91.1)	17 (8.9)
Sexual Abuse (Forced Contact)	188	187 (99.5)	1 (0.5)
Financial Exploitation	185	179 (96.8)	6 (3.2)

GeriHITSS$ responses indicated most participants (96.8%) denied experiencing physical abuse, while 3.2% reported some level of physical harm. Emotional abuse was reported by a small but notable proportion of respondents: 82.0% denying being insulted, whereas 11.6% and 5.3% indicated “sometimes” and “fairly often” emotional abuse, respectively. Similarly, 97.9% of participants reported they had never been threatened, with only 2.1% total experiencing some level of threatening behavior. Verbal abuse in the form of screaming or cursing was reported by 8.9% of respondents, while 91.1% denied such experiences. Forced sexual contact was the least reported form of abuse, with only one participant (0.5%) indicating such an experience. Financial exploitation was also relatively rare, with 96.8% denying any abuse and 3.2% reporting some level of financial abuse (Table 3). Out of 199 participants, 39 reported experiencing abuse of any type and at any level.

The 6-item GeriHITSS$ demonstrated acceptable internal reliability (α = .721), and the 4-item HITS showed comparable reliability (α = .753). The mean HITS score across all participants was 0.55 (SD = 1.63; range 0-12). The mean GeriHITSS$ score across all participants was 0.45 (SD = 1.35; range 0-12).

Scaled scores for GeriHITSS$ were not significantly correlated with age (*r* = -0.071, *p* = .352). Though women had slightly higher mean GeriHITSS$ scores (*m* = 0.60, SD = 1.93) than men (*m* = 0.46, SD = 1.09), this difference was not statistically significant (*t* = [1, 188] = - .566 *p* = .572, *d* = -.083). Similarly, no significant differences in GeriHITSS$ scores were observed between White and non-White participants (*t* [1, 187] = -.028, *p* = .978, *d* = -.004).

## Discussion

This pilot study provides preliminary evidence supporting the internal reliability of the GeriHITSS$ as a screening tool for elder abuse in family medicine outpatient settings. The prevalence of self-reported abuse identified in our sample was comparable to estimates reported in previous studies, suggesting that elder abuse remains a common yet often underrecognized problem among older adults.^1,3^

These findings indicate that the GeriHITSS$ may be useful in identifying individuals at risk for abuse in routine clinical practice while capturing abuse domains that are not routinely assessed by existing screening instruments. The GeriHITSS$ demonstrated strong internal consistency that was comparable to that reported for the original HITS instrument. This finding suggests that the addition of financial exploitation and sexual abuse items did not diminish the reliability of the measure and may enhance its relevance for older adult populations, in whom these forms of abuse are important but frequently undetected. The results therefore support the feasibility of expanding the original HITS framework to better address the unique risks faced by older adults.

Emotional abuse was the most commonly reported form of abuse in our study, consistent with prior research showing that psychological abuse is among the most prevalent forms of elder abuse and is often disclosed more readily than physical, sexual, or financial abuse.^17,18^ In contrast, reports of physical, sexual, and financial abuse were less frequent. While this may reflect lower prevalence rates, it may also indicate ongoing challenges in detection and disclosure, as older adults may be reluctant to report sensitive experiences because of fear, shame, dependency on caregivers, or concerns about potential consequences.

The lack of significant differences in HITSS$ scores across demographic variables, including sex and race, is notable. While prior studies suggest that elder abuse risk varies based on gender and socioeconomic factors, our results did not indicate any statistically significant disparities.^18^ The lack of observed differences may also reflect the relatively homogeneous study population and the limited statistical power of this pilot sample. Together, these findings support growing evidence that contextual factors, including caregiver relationships, social isolation, and available support systems, may play a more important role in elder abuse risk than demographic characteristics alone.

This study has several notable strengths. The use of a cross-sectional design within real-world primary care settings enhances the ecological validity of the findings, as data were collected during routine clinical visits. Recruitment from two family medicine clinics increases the generalizability of results across diverse outpatient populations. Importantly, the study population itself was racially and ethnically diverse, with no single group in the majority, allowing for examination of the tool’s performance across groups often underrepresented in prior research. The inclusion of both English and Spanish speaking participants further strengthens the cultural and linguistic applicability of the tool, while administration protocols, such as ensuring family members could not influence responses, promoted data integrity. Finally, the use of rigorous analytic methods, including internal consistency testing, mean comparisons, and effect size calculations, provides robust evidence for the reliability of the GeriHITSS$ in detecting abuse among older adults.

Despite its contributions, this study has several limitations. Reliance on self-reported data may have introduced response bias, as participants may have underreported abuse due to fear of retaliation or social desirability concerns. The number of eligible patients who declined participation and their reasons for refusal were not systematically recorded; therefore, potential nonresponse bias could not be assessed. The study’s cross-sectional design also limits the ability to determine causality between abuse risk factors and outcomes. Additionally, the study setting may influence results; factors such as different geographic locations or healthcare environments might yield varied patterns of abuse detection.

An important limitation of the screening tool itself is that it focuses primarily on active forms of elder abuse and does not assess passive forms of abuse such as neglect or abandonment. Consequently, a score of zero should not be interpreted as ruling out all forms of elder abuse, and clinical judgment remains necessary when evaluating vulnerable older adults. Furthermore, because the tool has undergone only preliminary testing within a single study sample and was not confirmed using an independent verification cohort, it should not yet be considered a comprehensive or standardized instrument. Rather, this study should be viewed as a pilot reliability study that provides initial evidence of the tool’s consistency and feasibility.

The Mini-Cog screening tool, used to assess cognitive function, may have excluded individuals experiencing abuse, particularly those with trauma-related cognitive dysfunction. This exclusion does not align with clinical practice, where patients with mild cognitive impairment are still evaluated for abuse risk. Future research should explore alternative screening strategies that do not inadvertently omit vulnerable populations.

The GeriHITSS$ tool was developed to meet essential criteria for screening effectiveness, offering a new approach for elder abuse detection. This study underscores the need for continued refinement of screening tools that comprehensively assess multiple abuse domains. From a health policy perspective, the findings suggest that a brief and reliable screening tool such as the GeriHITSS$ could support the systematic identification of elder abuse in primary care settings. Incorporating elder abuse screening into routine outpatient care may facilitate earlier intervention, improve referrals to social and protective services, and inform the development of policies aimed at strengthening elder abuse prevention and response efforts. As the population ages, scalable screening approaches may help healthcare systems better identify vulnerable older adults and allocate resources to those at greatest risk.^19^

Additional research is needed to validate the tool across diverse populations and settings and to determine an appropriate cut-off score for clinical intervention. This study evaluated internal consistency reliability but did not assess criterion validity through comparison with an established elder abuse screening instrument or comprehensive clinical assessment. As a result, the sensitivity, specificity, and optimal cut-off score of the GeriHITSS$ remain unknown and should be examined in future validation studies. Future studies should also explore incorporating neglect, abandonment, and other forms of elder abuse to improve the comprehensiveness of the screening process. Future work should focus on further development and integration of such tools into routine geriatric care, a critical priority given the significant health consequences associated with elder abuse.

## Conclusion

The GeriHITSS$ demonstrated good internal consistency in a sample of older adults receiving care in family medicine outpatient clinics, supporting its potential as a brief screening tool for elder abuse. By expanding the original HITS instrument to include financial exploitation and sexual abuse, the GeriHITSS$ addresses forms of abuse that are frequently underrecognized in clinical practice. These findings provide preliminary evidence supporting the feasibility and reliability of the instrument in outpatient geriatric populations. Additional research is needed to establish its validity, determine clinically meaningful cut-off scores, evaluate diagnostic performance, and assess its applicability across diverse populations and healthcare settings.

## Data Availability

The datasets generated and analyzed during the current study are available from the corresponding author on request, and requests will be reviewed by all authors.*
